# Retraction: The turnaway study: A case of self-correction in science upended by political motivation and unvetted findings

**DOI:** 10.3389/fpsyg.2022.1130026

**Published:** 2022-12-26

**Authors:** Frontiers Editorial Office

The journal retracts the 17 June 2022 article cited above.

Following publication, undisclosed competing interests were brought to our attention, which undermined the objective editorial assessment of the article during the peer review process.

Frontiers conducted a post-publication assessment of the article, including consulting with independent expertise, which concluded that the article does not meet the standards of publication of Frontiers in Psychology.

This retraction was approved by the Field Chief Editor of Frontiers in Psychology and the Chief Executive Editor of Frontiers. The authors did not agree with this retraction.

